# Supplementary material for: The development of a novel diagnostic PCR for Madurella mycetomatis using a comparative genome approach
Source: PLoS Negl Trop Dis. 2020 Dec 16;14(12):e0008897. doi: 10.1371/journal.pntd.0008897 (PMC7743967; doi:10.1371/journal.pntd.0008897)
Supplement: Attachment — Submitted filename: PLOSNTD Letter to reviewer .docx [file pntd.0008897.s001.docx]

**Reviewer's Responses to Questions**

Dear PLoS and reviewers,

I thank you for your time and effort spent in reviewing this manuscript titled ‘The development of a novel diagnostic PCR for Madurella mycetomatis using a comparative genome approach’. In this document we have addressed the issues brought up by reviewers and provided answers accordingly.

We have tried our outmost best in answering the questions raised, you may find the answers below.

Sincerely,

Wilson Lim

(First author and on behalf of all authors)

**Reviewer 1**

**1. The fungal isolates tested. It isn't clear how these were all speciated prior to testing. Was this carried out by molecular techniques (please state how) - This information should be included in the paper.**

All isolates used in this study were identified to the species level on the basis of morphology, polymerase chain reaction (PCR)-based restriction fragment length polymorphisms, and sequencing of the ITS regions. This information is included in the manuscript under the sub-section ‘fungal isolates and DNA isolation’ of the materials and methods section.

**2. It is a pity that some mycetoma agents have not been used for comparison eg Scedosporium apiospermum as other non-pigmented fungi have been included. Was this an oversight?**

We initially focused on fungi closely related to *Madurella mycetomatis* and then included more black-grain mycetoma causative fungi and also those that are common cause of lab-contaminations. Therefore, *Scedosporium apiospermum* was not included. We have now included the data on *S. apiospermum* in Table 1 and in the manuscript. The new primers Mmy-Fw and Mmy-Rv did not cross react to *S. apiospermum* and is specific for *M. mycetomatis.* Thank you for the suggestion as this indeed strengthens the manuscript.

**3. In the discussion the authors refer to the *in silica* assay. Can you expand on this ?**

The authors sequenced seven *Pseudoperonospora cubensis* isolates and ten *Pseudoperonospora humuli* isolates and determined sequences that are specific to *P. cubensis* but not in *P. humili* *in silica*. By using this approach, they were able to determine the specificity of a large number of sequences and reduce the number of candidate genes to be validated in the wet lab using PCR. We were unable to compare sequences between *M. mycetomatis* isolates *in silico* because at the time of data analysis, only one *M. mycetomatis* genome was available. We were also not able to compare *M. mycetomatis* sequences between *M. fahalii, M. tropicana* and *M. pseudomyetomatis* because none of their genomes were sequenced.

The sentence “their organism of interest while reducing the number of diagnostic candidates to validate with PCR” was added on line 203.

**4. It would be helpful if the authors would say where they see the place of reference centres for mycetoma diagnosis. How many are there and do we need more ?**

The main reference center is the WHO Collaborating Center on Mycetoma in Khartoum, Sudan. More reference centers are needed and ideally one for every region in endemic areas. With the establishment of the Global Mycetoma Working Group in 2016, a surveillance unit was set up consisting of expert from different countries from endemic areas. This surveillance unit is tasked with gathering data of mycetoma cases and to also raise awareness of this disease. With data from surveillance, the global burden for mycetoma will be understood and then more reference centers can be established according to the demography.

To clarify this, we have added the following sentence in the discussion line 214: “We recommend reference centers such as the WHO collaborative Mycetoma Reference Center in Khartoum, Sudan and yet to be established reference laboratories in other endemic countries to use the new PCR primers Mmy-Fw and Mmy-Rv to identify *M. mycetomatis* to the species level”

**Reviewer 2**

**1. The manuscript by Lim et al describes the development of a novel PCR assay for the specific detection/identification of cases of eumycetoma caused by Madurella mycetomatis, based on a genome-wide comparative approach.**

**A series of potential gene targets for PCR were identified in putative coding regions throughout the M. mycetomatis genome, PCR primers were designed and tested against a panel of 60 M. mycetomatis isolates plus a range of sibling species in Madurella, other agents of eumycetoma, and a variety of less related filamentous fungi.**

**One primer pair was selected due to its high specificity, and was shown to be slightly less sensitive that the previously described spscific PCR (which has since been shown to cross-react with another Madurella species.**

**A major question that should be addressed is whether this novel PCR is sufficiently sensitive to be used in diagnosis directly from clinical specimens eg fungal grains, or whether it will only be useful when working with pure cultures. I would encourage the authors to address this in the current manuscript as it would substantially strengthen the study.**

To validate the selected PCR primers, we are currently performing a large study directly on grains in the mycetoma research center. In that study we will validate PCR versus culture and histology. This will be performed on grains obtained from a surgical biopsy. To validate the PCR method, the ITS based primers and the newly developed primers here will be used. This will give the answer to the question raised above. Preliminary data on clinical specimen testing has shown that our primers is sensitive enough to be used in diagnostics from DNA obtained directly from grains. We have added these results into the manuscript from line 181 onwards and in figure 2. Thank you for the suggestion as this has greatly improved the manuscript.

**2. The English usage is a little poor in places, and grammatical issue should be addressed on lines 35 (were not was); 50 (disappointing? is not clear); 54 (cause not causes); 57 (delete "a"); 57 (suggest: that we recommend"); sentence starting line 78 (developed is repeated)108 (using substituting?);127 (PCR reactions contained); 129 (were as follows); 132 (was this final stage really 10 seconds?); Table 2 (Madurella tropicana and spelling of Trematosphaeria); 154 Primer sets 4, 5 and 12 only amplified ...); 189 (delete "a")**

Thank you for the comments and suggestions. The grammatical errors pointed out were addressed accordingly.
